# Towards Circular Economy by the Valorization of Different Waste Subproducts through Their Incorporation in Composite Materials: Ground Tire Rubber and Chicken Feathers

**DOI:** 10.3390/polym14061090

**Published:** 2022-03-09

**Authors:** Xavier Colom, Javier Cañavate, Fernando Carrillo-Navarrete

**Affiliations:** 1 Department of Chemical Engineering, Universitat Politècnica de Cataluna—BarcelonaTECH, Colom 1, 08222 Terrassa, Barcelona, Spain; xavier.colom@upc.edu (X.C.); fernando.carrillo@upc.edu (F.C.-N.); 2 Institut d’Investigació Tèxtil i Cooperació Industrial de Terrassa (INTEXTER), Universitat Politècnica de Catalunya (UPC)—Barcelona TECH, Colom 15, 08222 Terrassa, Barcelona, Spain

**Keywords:** waste, composites, tire rubber, chicken feathers, acoustic properties

## Abstract

Incorporation of residua into polymeric composites can be a successful approach to creating materials suitable for specific applications promoting a circular economy approach. Elastomeric (Ground Tire Rubber or GTR) and biogenic (chicken feathers or CFs) wastes were used to prepare polymeric composites in order to evaluate the tensile, acoustic and structural differences between both reinforcements. High-density polyethylene (HDPE), polypropylene (PP) and ethylene vinyl acetate (EVA) polymeric matrices were used. EVA matrix defines better compatibility with both reinforcement materials (GTR and CFs) than polyolefin matrices (HDPE and PP) as it has been corroborated by Fourier transform infrared spectroscopy (FTIR), termogravimetric analysis (TGA) and scanning electron microscopy (SEM). In addition, composites reinforced with GTR showed better acoustic properties than composites reinforced with CFs, due to the morphology of the reinforcing particles.

## 1. Introduction

Nowadays, one of the greatest concerns of humanity is the huge amounts of waste that are produced year after year around the world. Therefore, strategies for the valorization of wastes or by-products based on recovering and recycling have been developed during the last years in order to reduce the negative environmental impact caused by the use and transformation of resources [1,2]. There are many kinds of waste, but special attention is paid to materials that are not biodegradable, such as plastics [3,4], or to residua that, even being biodegradable, are produced in great quantities and can cause massive environmental impacts, including climate-harming emissions by illegal dumping or burning, such as crop wastes, eggshells, chicken feathers, crustacean shells and others [5].

The transition from waste disposal to a circular economy is in evolution. Consequently, many alternatives of waste valorization are currently under research and development. Hence, research all over the world focuses on the development of strategies for recycling [6] and upcycling [7] plastic wastes and, in the case of biowastes, on the advance of integrated biorefinery concepts or the production of basic chemicals and specialized fibers among others [5].

In parallel to the aforementioned approaches, several research groups are working with waste materials to obtain newly added value materials [8,9]. In this regard, a commonly used approach is to recycle wastes by mixing them with polymeric matrices to obtain new composite materials. On one hand, the use of natural fibers, from plants (jute, sisal, flax, etc.) or animals (wool, hair, feathers, etc.), to prepare polymer composites have gained attention during the last decades [10,11] and it has expanded considerably in some sectors, such as in automotive industry [12]. However, natural fibers are mainly hydrophilic, hindering the interfacial adhesion between the fiber and the hydrophobic polymeric matrices resulting in composite materials with weak mechanical and physical properties unless some treatments are performed on the fibers [13]. On the other hand, non-biodegradable wastes have also been proposed as reinforcements or fillers for the preparation of polymeric composites [14,15].

In this regard, tire rubber has been proposed as filler or reinforcement in order to manufacture composites with thermoplastic, thermosets and rubber matrixes with interesting tensile, electrical, or acoustical properties [16,17,18]. This approach allows the recovery and reuse of part of a tire rubber avoiding their disposal in landfills. Following the same idea, there are several interesting biogenic abundant and biodegradable wastes that would end in a landfill unless they were reused somehow, for example, by mixing the biogenic waste with polymeric matrices. Among the several biogenic wastes generated by industrial processes, chicken feathers (CFs) are generated in large amounts and they do not have any practical application, so they are a potential candidate for developing composite materials [19].

The main challenge for composite preparation by using both non-biodegradable and biodegradable wastes is to investigate how properties and development of these materials are influenced by the compatibility between the composite components since the fiber-matrix interaction can significantly affect the final macroscopic properties of the composite product. In the case of tire rubber and CFs, when they are blended with polymeric matrices (HDPE, PP, EVA, etc.), the fiber-matrix compatibility is expected to be low [19,20]. One way to increase the compatibility between both components is to carry out a pretreatment of the waste. For example, acid pretreatment with nitric and/or sulfuric acids due to chemical reactions produces a microporous surface that improves mechanical adhesion. The chemical attack leads to cavities that develop porosity on the waste particles, allowing a good interlocking with polymeric matrices [20]. In addition, grafting of polymers and compatibilizing agents have been used on tire powder to obtain useful materials, improving the interfacial adhesion [21]. In the same way, pretreatments of CFs have been proposed to prepare composite materials with enhanced mechanical properties [19]. In any case, the nature of each particular waste will have an impact on the final fiber-matrix compatibility and it is worth studying. Consequently, a comparative study is proposed in this manuscript in order to evaluate the inherent differences in compatibility and processing when using either GTR or CFs without the help of chemical compatibilization pretreatments.

Taking into account these premises, the main purpose of this study is to compare elastomeric (Ground Tire Rubber or GTR) and biogenic waste (chicken feathers or CFs) composite materials in order to understand the tensile and structural differences between both and which is the best for specific industrial applications. The aim is also to provide examples of two very different residua that can be useful in order to undertake related cases promoting a circular economy approach. High-density polyethylene (HDPE), polypropylene (PP) and ethylene vinyl acetate (EVA) polymeric matrices were used. Once the composites were prepared, mechanical and structural characterization were carried out by using tensile test, scanning electron microscopy (SEM), Fourier-transform infrared spectroscopy (FTIR) and thermogravimetric analysis (TGA) techniques to find a relationship between the macroscopic properties, i.e., tensile strength, and microstructure. In addition, the acoustical properties of the materials were determined to evaluate the viability of using them for sound absorber applications. Finally, the properties of the composites were compared in order to discern which type of waste provided better performance.

## 2. Materials and Methods

### 2.1. Materials

High-density polyethylene (HDPE, ALCUDIA^®^ 4810-B, Repsol, Tarragona, Spain) with a melt flow index of 1.35 g/min and density of 960 kg/m^3^, Polypropylene (PP Isplen^®^ 099 K2M, Repsol, Tarragona, Spain) with a melt flow index of 1.15 g/min and density of 913 kg/m^3^ and Ethylene vinyl acetate (EVA ALCUDIA^®^ PA 539, Repsol, Tarragona, Spain) with a melt flow index of 1.18 g/min and density of 937 kg/m^3^, were used in this study.

Ground Tire Rubber (GTR) with a size lower than 400 µm was supplied by GMN Company from Maials (Lleida, Spain) and were used without treatment. 

Chicken feathers (CFs) were kindly supplied from a slaughterhouse located in Catalonia (Spain). CFs from slaughterhouses are unstable, unsafe and biodegradable, so pretreatment is mandatory to stabilize and sanitize the waste. Therefore, the CFs were first frozen at −20 °C and subsequently washed in a washing machine at 35 °C with a 3300 ppm H_2_O_2_ solution (hydrogen peroxide 35% *w*/*v*, Chem-Lab NV, Zedelgem, Belgium), in a 5/1 (*vol/wt*) liquor ratio for 50 min. After that, CFs were dried in an air oven at 60 °C for 24 h. Cleaned CFs were later chopped with a shredder (Retsch SM100, Haan, Germany) until each particle size was 2 mm or less. Finally, the CFs were air-dried at 105 °C for 4 h and kept under a dry atmosphere (desiccator) just before the composites were compounded.

### 2.2. Composite Preparation

Composite specimens were obtained by mixing the ground and dried particles of CFs and GTR previously prepared, with HDPE, PP and EVA matrices. Different compositions were studied: 5, 10, 20, and 40% *w*/*w* and controls of pure HDPE, PP and EVA were used as references.

The components were mixed using a Brabender mixer type W 50 EHT PL (Brabender^®^ GmbH & Co. KG, Duisburg, Germany) heated at different temperatures for each matrix: 170 °C for PP; 160 °C for HDPE and 120 °C for EVA, respectively, and at a mixed speed of 50 rpm. The HDPE, PP and EVA matrices were melted for a minute and then, the particles (GTR or CFs) were added and mixed for a period of 5 min.

These blends were then consolidated in a hot plates press machine type Collin Mod. P 200E (Dr. Collin GmbH, Maitenbeth, Germany) forming square sheets, measuring 160 × 160 × 2.2 mm^3^. Consolidation was carried out at a pressure of 100 kN for 5 min using temperatures of 180 °C, 150 °C and 100 °C for PP, HDPE and EVA composites, respectively. Finally, the square sheets were cooled under pressure using cool water.

Test samples were properly dumbbell shaped according to the ASTM 412 specifications to carry out tensile test measurements [22].

### 2.3. Tensile Tests

The tensile test measurements were carried out in an Instron 3366 universal machine (Instron, High Wycombe, UK). The fabricated composites can be considered isotropic since the GTR particles are meanly spherical and the chicken feathers, although they are fibers, are randomly oriented, so loading direction was not taken into account. The testing speed was 20 mm/min at room temperature. The samples cross-sections were 6.1 × 2.2 mm^2^. Young’s modulus, tensile strength, elongation at break and toughness were calculated using Bluehill version 2 software. Five replicate samples were analyzed for each test and average and standard deviation percentages were calculated.

### 2.4. Structural Characterization by Fourier Transform Infrared Spectroscopy (FTIR)

The structural properties have been obtained using FTIR by means of a Nicolet Avatar spectrometer with CsI optics. Samples of the powdered rubber (400–600 µm average particle size) and powdered chicken feathers were ground and dispersed in a matrix of KBr (9 mg in 300 mg KBr), followed by compression at 167 MPa to consolidate the formation of the pellet.

### 2.5. Characterization by Scanning Electron Microscopy (SEM)

SEM was used to qualitatively examine the fracture surface of the samples broken by the mechanical tests to study the compatibility at the different samples reinforced by CFs or the GTR interface. Several images of the samples were taken in a JEOL 5610 microscope at an accelerating voltage of 30 kV and a working distance of 6 mm. Previously to the observations, the samples were covered with a fine layer of gold-palladium in order to increase their conductivity.

### 2.6. Characterization by Thermogravimetricy Analysis (TGA)

Thermal treatments were performed by means of TGA. TGA was performed in a TG/SDTA851 Mettler Toledo equipment at 10 °C/min heating rate in N_2_ atmosphere, to obtain the TGA degradation process curve. The temperature range was from 30 to 600 °C, the typical degradation range of the thermoplastic matrix composite. A mass in a range of 10–12 mg of sample was analyzed in order to guarantee sample homogeneity that is of particular importance for analytical techniques, such as TGA.

### 2.7. Characterization of Acoustic Properties by Impedance Tube

The acoustic properties were measured using a two microphone impedance tube Brüel & Kjaer type 4206 ((Brüel & Kjaer, Virum, Denmark) in the frequency range 100–6500 Hz, according to the specification ASTM E 1050, which describes the standard test method for impedance and absorption of acoustical materials using a tube, two microphones and a digital frequency analysis system. Cylindrical samples (2.2 mm of thickness) were prepared by cutting the material and then submitted to a plane sound wave. The sound pressures were measured at the same time in two microphone positions and the relationship between the acoustic energy that is absorbed by the material and the total incident energy resulted in the normal incidence sound absorption coefficient (α).

## 3. Results and Discussion

### 3.1. Mechanical Properties

**Figure 1:**
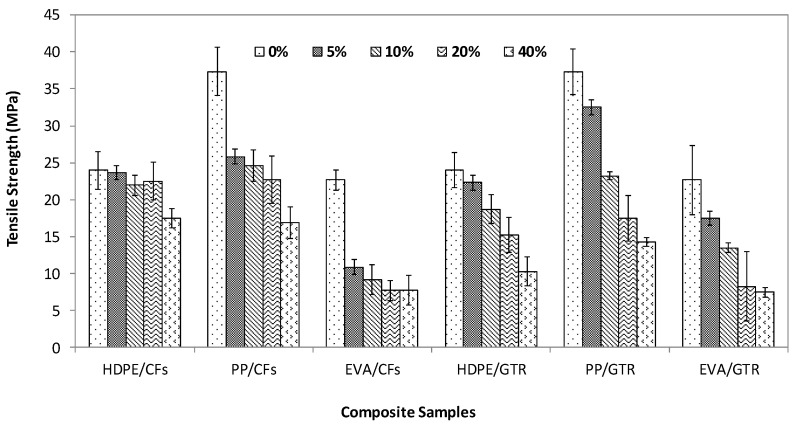
Tensile strength of the polymeric composites at compositions between 0 and 40% *w*/*w*.

**Figure 2:**
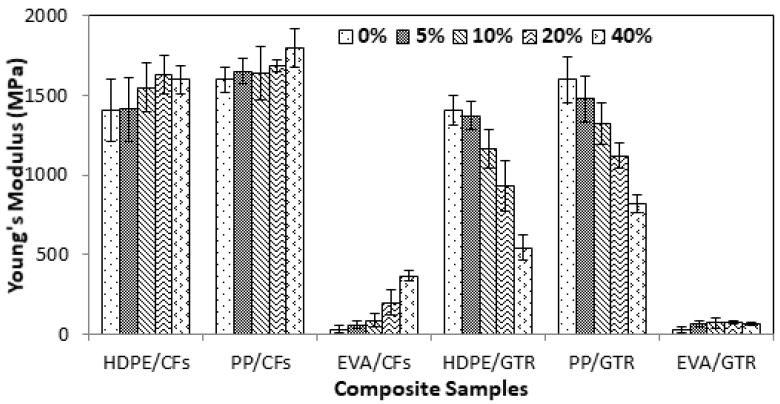
Young’s Modulus of the polymeric composites at compositions between 0 and 40% *w*/*w*.

**Figure 3:**
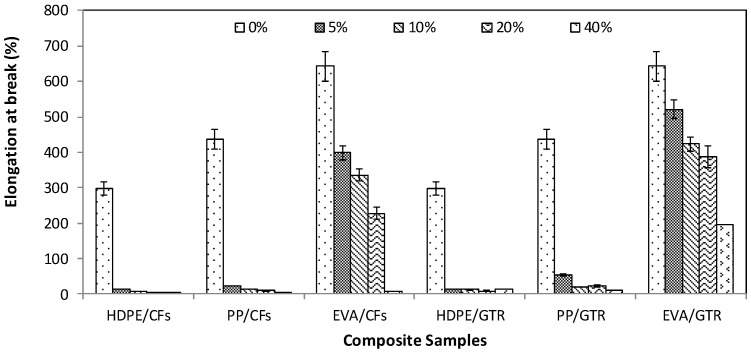
Elongation at break of the polymeric composites at compositions between 0 and 40% *w*/*w*.

Figure 1, Figure 2 and Figure 3 show the evolution of tensile strength, Young’s Modulus and elongation at break of two different kinds of composite made by polyolephynic matrix and two waste reinforcements (GTR and CFs).

The values of tensile strength showed similar behavior for all composites (Figure 1). All of them decreased as a function of reinforced content, although the evolution is quite different for each one. The composites reinforced with GTR had a continuous decrease; meanwhile, the composites made by CFs had a decrease in two steps (contents of 5% and contents from 5% to 40%). Only with 5% of CFs, the value of tensile strength decreased 14.2% for the HDPE/CFs; 30% for the PP/CFs and 56% for EVA/CFs and after that the values decreased more slightly for the different contents of CFs. The values for the composites reinforced with GTR had a continuous evolution from pure matrix to compositions of 40% showing a total decrease of 49.5% for HDPE/GTR; 62.5% for PP/GTR and 66.3% for EVA/GTR.

According to the results shown in Figure 1, it was confirmed that the tensile strength of PP matrix composites was higher than other thermoplastic matrices used. This fact was very evident in CFs reinforcement, although it also took place in GTR reinforced composites.

The differences in behavior between both kinds of reinforcements are due to their dissimilar chemical and morphological nature and their compatibility with the matrices, which defines more or less lack of interfacial adhesion. CFs are hydrophilic compared to the highly hydrophobic nature of the GTR and the morphology of the chopped chicken feathers is long and sharp meanwhile the GTR has essentially irregular polyhedron geometry.

Young’s modulus of CFs reinforced composites increased slightly as a function of the percentage of CFs (Figure 2). The most important increase took place in composites of EVA/CFs with an increase of 1200% whereas for HDPE/CFs and for PP/CFs the increase was only 13.7 and 12.4%, respectively. Instead, Young’s modulus in composites reinforced by GTR decreases significantly. This means that the compatibility between thermoplastic matrices was better in CFs than in GTR. The Young’s Modulus of CFs composites increased slightly at low contents of CFs, except in EVA/CFs, where the increase was significant. This is due to its ability to interact with CFs. The presence of GTR with a maximum size of 200 µm increased only the rigidity of EVA/GTR composites since the particle-matrix compatibility was not good enough, as has been demonstrated in previous works [23,24]. Only, in EVA/GTR Young’s modulus increased 157% due to the different chemical composition of ethylene vinyl acetate with respect to HDPE and PP, with ester and carbonyl groups that are more reactive than methyl and methylene groups of HDPE and PP.

In all cases (CFs and GTR based composites), mechanical properties depend also on the dispersion of the reinforcement in the HDPE, PP and EVA matrices, since the CFs and GTR particles are responsible for the decrease in the deformation capacity in the elastic zone. Thermoplastic matrices provide ductility whereas the reinforced particles exhibit brittle behavior with a subsequent loss of toughness to the composite material. The elongation at the breaking point mainly depends on the reinforced particle content, obtaining better behavior for composites with EVA matrix (Figure 3). This mechanical property decreased from 297% to 14% (HDPE/CFs), 436% to 23% (PP/CFs), 642% to 401% (EVA/CFs), 297% to 13.4% (HDPE/GTR), 436% to 53%(HDPE/GTR) and 642% to 521% (HDPE/GTR) when incorporating only 5% of reinforced particles. When reinforced particles increase until contents were higher than 20% *w*/*w*, there was not a significant difference of elongation for a polyolephynic matrix. For all reinforcement contents, EVA matrix composites showed a better performance. As shown in Figure 3, the elongation at break using EVA as a matrix in composites with 20% *w*/*w* of reinforcement had important differences using GTR or CFs.

### 3.2. SEM Characterization of Interfacial Adhesion

**Figure 4:**
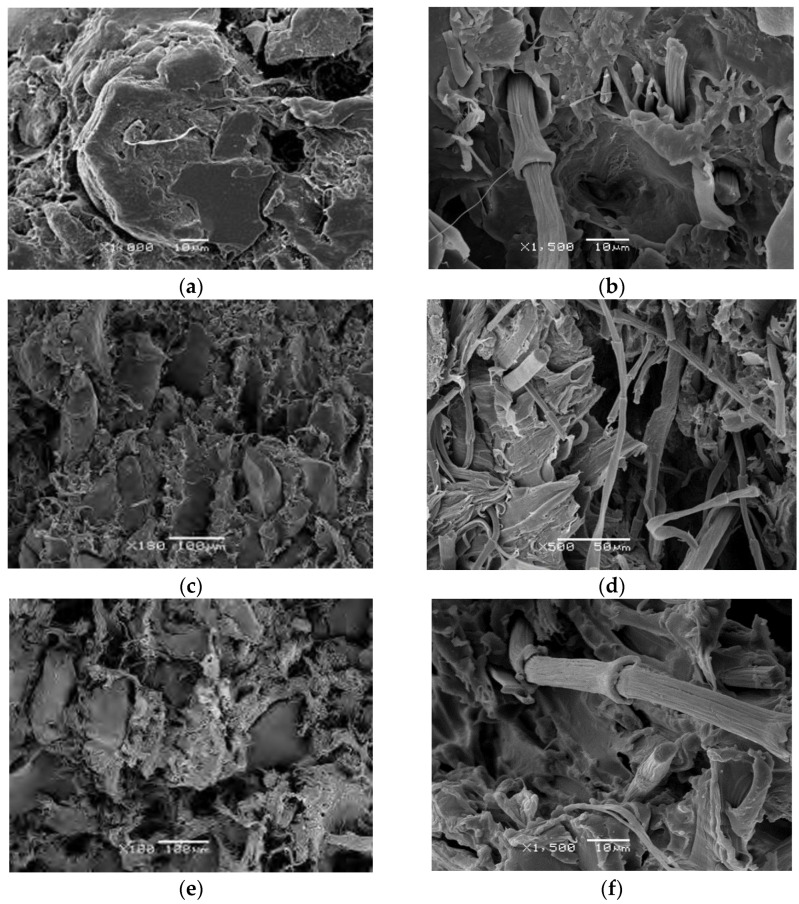
Scanning electron microscopy of the fracture surfaces of CFs and GTR based composites at composition of 40%: (**a**) HDPE/GTR, (**b**) HDPE/CFs, (**c**) PP/GTR, (**d**) PP/CFs, (**e**) EVA/GTR and (**f**) EVA/CFs.

As shown in Figure 4, SEM micrographs of fracture surfaces of different composites (all containing 40% of CFs and GTR particles), clearly indicate that the differences in microstructure of the various composites are significant. First of all, the samples containing GTR particles (Figure 4a,c,e) show some cracks and pores big enough to be observed at this level of magnification. The GTR particle is unlinked to the matrix, as can be observed by the deep voids around its contour. The rubber seems to be resting on the thermoplastic matrices, without being properly attached to them. On the other hand, the matrices have been strained and deformed plastically, independently of the GTR, which remains unchanged. The samples containing CFs (Figure 4b,d,f) show fibers cleanly extracted from the matrices. No residues or portions of matrix material have adhered to the fiber surface. There are also voids subsequent to the pull-out of CFs that can be observed on the surface. In EVA matrices, although there is fiber pullout, some of them are coated with the EVA matrix. It can also be observed that the break of the composites takes place mainly by shear yield and tearing. The differences between the failure surface of different matrix composites are attributed to the different chemical natures of the matrices and different adhesion mechanisms.

The SEM images corroborate that the interaction is considerably less intense than the cohesion forces of the matrices.

### 3.3. FTIR Characterization

**Figure 5:**
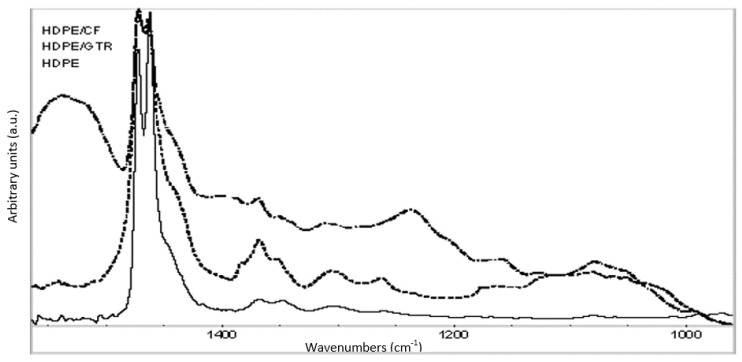
FTIR spectra of HDPE matrix and composites HDPE/GTR40 and HDPE/CFs40.

Results from FTIR analysis indicated that only a weak adhesion is expected for GTR and CFs based composites, corroborating the results of the SEM characterization. Figure 5 compares the spectral evolution of HDPE/CFs and HDPE/GTR composites, respectively. The main differences between both composites are: (i) the doublet 1464/1474 cm*^−^*^1^, (ii) the peak of 1370 cm*^−^*^1^ and (iii) the band at 1635 cm*^−^*^1^, assigned to –CH_2_–, –CH_3_ and water absorption, respectively. Analyzing the band at 1635 cm*^−^*^1^, it can be seen that the highest absorbance value corresponds to the CFs reinforced composites and the lowest to the GTR reinforced composite. The different patterns of this band, in relation to the types of reinforcement used, are due to the hydrophilic nature of the keratin fiber. Another difference between both kinds of HDPE matrix composite materials is the band’s relation 1464/1474 cm*^−^*^1^ associated with the crystalline phase of HDPE. The spectra show that the evolution of the doublet in the HDPE matrix is larger in CFs than in GTR, and this means that the CFs create vibrational perturbations originated by the peptidic bonding in the HDPE backbone, affecting the degree of crystallinity in the matrix of CFs composite. However, these structural changes do not significantly improve the compatibility between the matrix and CFs to obtain composites with improved tensile properties. The band at 1370 cm*^−^*^1^, assigned to tensile stretch in methyl groups is higher in GTR composites than in CFs composites and overall HDPE matrix. This is due to the composition of GTR with several elastomers with methyl groups (i.e., natural rubber, polybutadiene, styrene-butadiene rubber).

**Figure 6:**
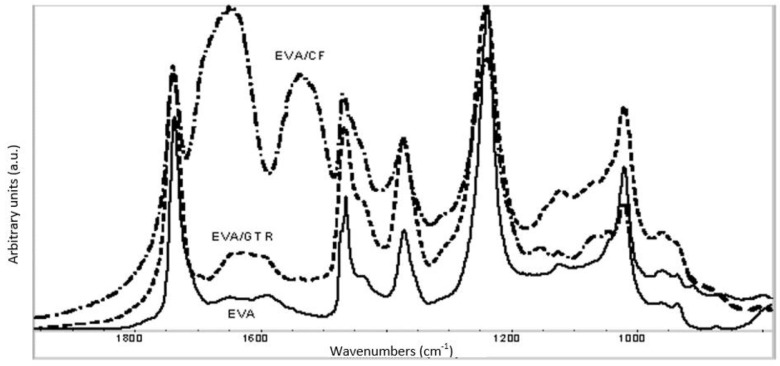
FTIR spectra of EVA matrix and composites EVA/GTR40 and EVA/CFs40.

Figure 6 shows the spectra of the EVA matrix and composite EVA/CFs and EVA/GTR. The comparative analysis of different spectra shows that in composite EVA/CFs, there is a difference in the maximum absorption of the band assigned to the carbonyl group of acetate component (1755 cm^−1^), which interacts with the amine group (1537 cm^−1^) moving the carbonyl group to a higher frequency. The band at 1150 cm^−1^ assigned to the COC group is another difference between both spectra composites. This band appears in the spectra of EVA/CFs and it is due to the interaction that take place between the acetate group of EVA and the queratinic groups of CFs.

This observation allows us to state that these two components present the best compatibility. As we can observe in the next section, these results are according to obtained mechanical properties of EVA/CFs, where all tensile properties were higher in value in EVA/CFs composites than in the materials obtained by the two other polyolephynic matrices.

**Figure 7:**
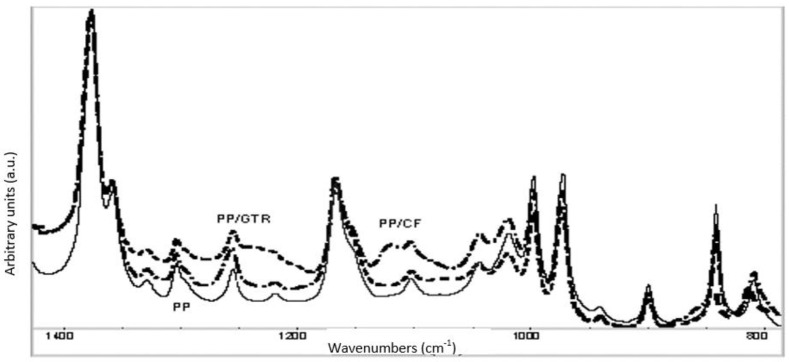
FTIR spectra of PP matrix and composites PP/GTR20 and PP/CFs20.

Figure 7 shows the spectra of the PP matrix and composite PP/CFs and PP/GTR. Analyzing the spectra comparatively, neither changes in the absorption bands nor band shifts were observed, verifying the lack of interaction between the particles and the matrix.

### 3.4. Thermogravimetric Analysis (TGA)

**Figure 8:**
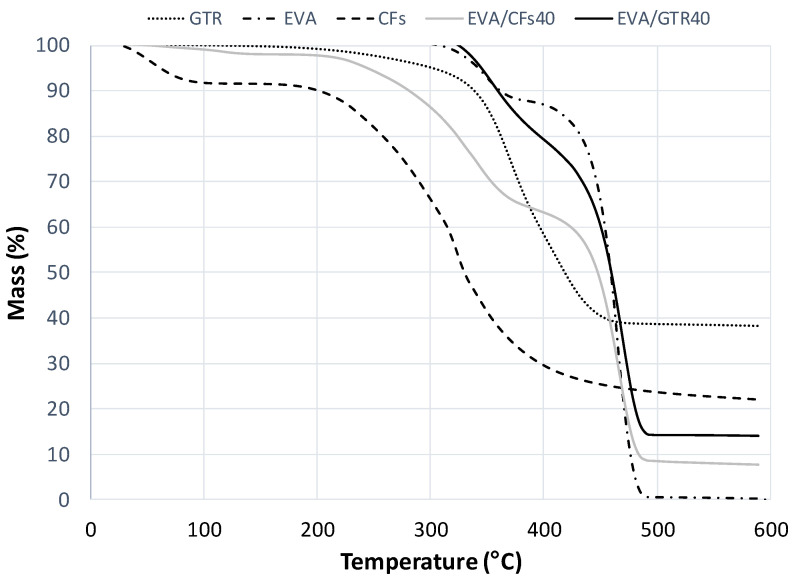
Thermograms of EVA/GTR and EVA/CFs composites at 40% composition and their corresponding components separately (EVA, GTR and CFs).

Figure 8 shows the thermograms of EVA/GTR and EVA/CFs composites and their corresponding components separately (EVA, GTR and CFs). The thermal behavior of CFs can be described in three main steps which are consistent with what has already been published by Tesfaye et al. [25]. In the first step, a loss of moisture was observed in the 30–200 °C temperature range. A second step was observed in the 200–360 °C temperature range corresponding to the partial decomposition of feather fractions that particularly consists in the denaturation of peptide bridges and protein chain linkages. In the third step, the feather fractions were decomposed from 360 °C to 550 °C. The residua obtained for the CFs (~20%) has been attributed to the inorganic components of the feathers [26]. For the GTR reinforcement, the thermogram showed the first loss of weight which starts at 280 °C corresponding to the release of volatile hydrocarbons and then continues until 350 °C. The second stage describes the release of the rest of the hydrocarbons with higher degradation temperatures (natural rubber, butadiene rubber, styrene butadiene rubber) until reaching a final mass that is 30–40% of the initial one [27]. The residua obtained for the GTR in the N_2_ atmosphere are composed mainly of carbon and SiO_2_ [28]. On the other hand, the EVA matrix showed a two-step thermal degradation process. The first stage, completed at around 370 °C, describes the deacetylation process in the vinyl acetate fraction. The second stage has been identified as complete chain scission of the residual main chain (within the interval of 380–480 °C) [29]. The comparative study of the thermal behavior of both composites allowed us to say that the first step of the thermograms (assigned to the decomposition of vinyl acetate of EVA) is completely different. The first step extension of EVA/CFs was higher than for EVA/GTR and this means that CFs interact with the EVA matrix. Analyzing both composites it can see that EVA/CFs had more compatibility than EVA/GTR. 

**Figure 9:**
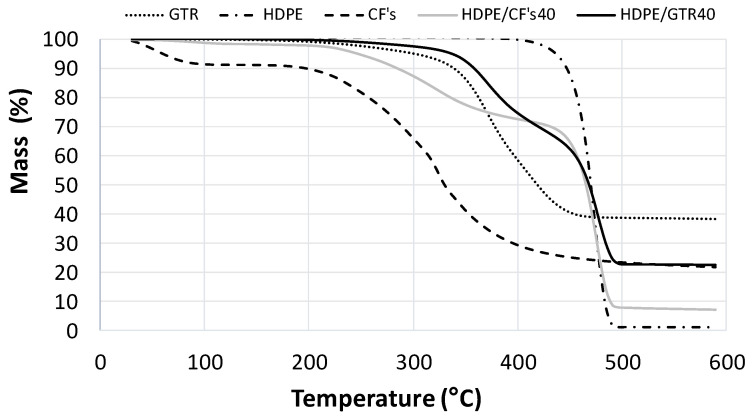
Thermograms of GTR/HDPE and CFs/HDPE composites at 40% composition and their corresponding components separately (EVA, GTR and CFs).

**Figure 10:**
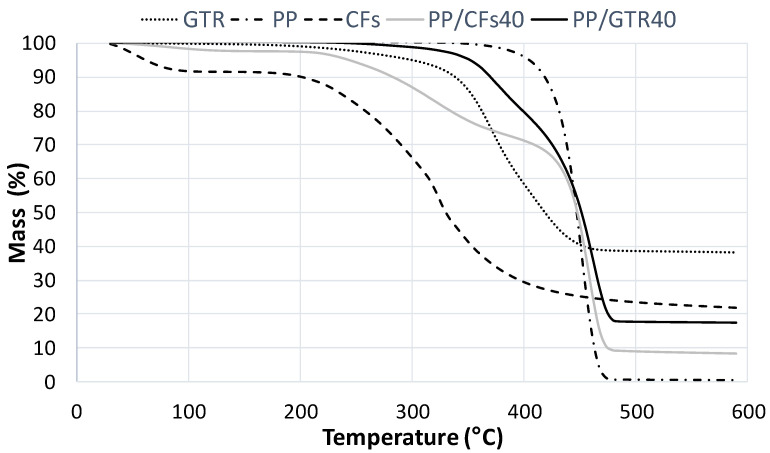
Thermograms of GTR/PP and CFs/PP composites at 40% composition and their corresponding components separately (EVA, GTR and CFs).

Figure 9 and Figure 10 show the thermograms of both kinds of composite using HDPE and PP as a matrix, respectively. The loss of HDPE and PP mass occurred in a single-stage degradation process that occurred over the temperature range of 400–500 °C [30,31]. Comparing the thermograms of both kinds of composites it can be concluded that the interaction between polyolephynic matrix (HDPE and PP) is higher with CFs reinforcement than GTR. In both cases, the first step that relates to reinforcement degradation is larger in CFs than GTR, which means that the degree of interaction is lower in GTR than CFs.

### 3.5. Acoustical Characterization

**Figure 11:**
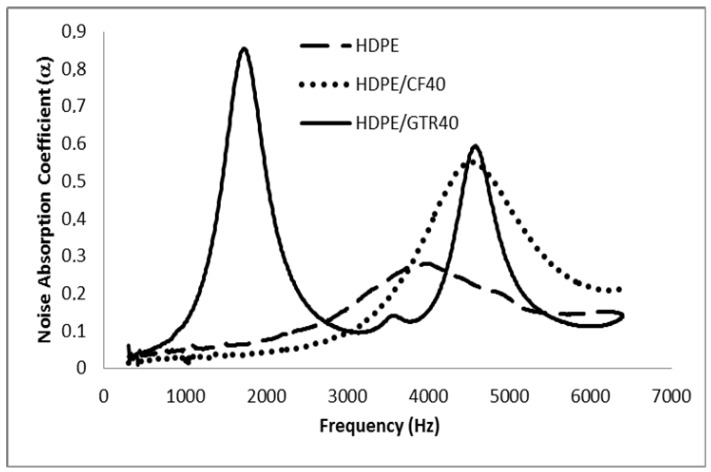
α values for HDPE and GTR/HDPE and CFs/HDPE composites at 40% composition.

**Figure 12:**
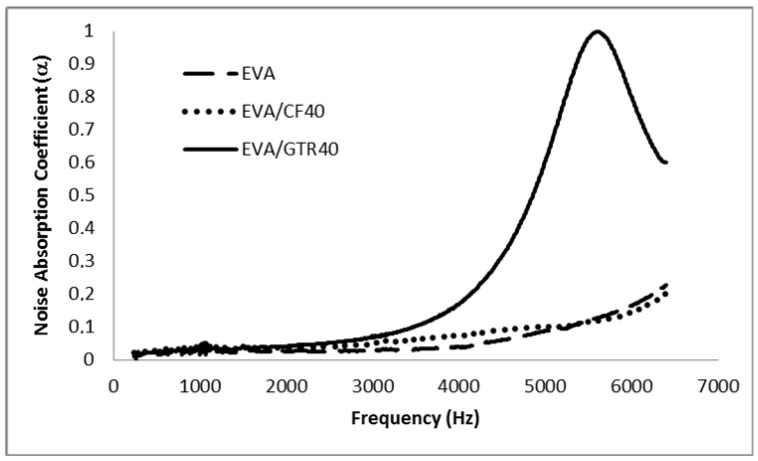
α values for EVA and GTR/EVA and CFs/EVA composites at 40% composition.

**Figure 13:**
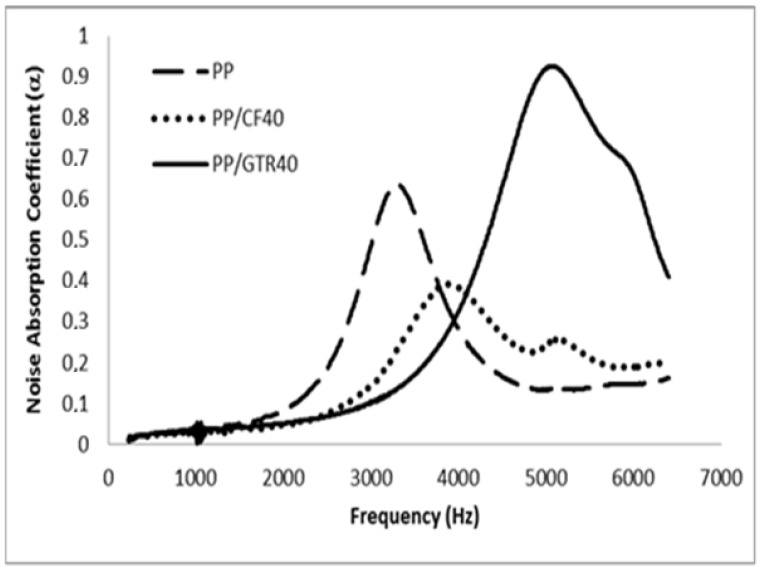
α values for PP and GTR/PP and CFs/PP composites at 40% composition.

The measured sound absorption coefficients, in 1/3 octave bands, of the composites samples reinforced with 40% of GTR and 40% of CFs are presented in Figure 11, Figure 12 and Figure 13. Only the HDPE/GTR40 show good results at low frequencies and mainly all the samples have acceptable results above 2500–3000 Hz. Figure 11 shows the absorption coefficient of the HDPE matrix and HDPE/CFs and HDPE/GTR. The results show that composite samples have a better noise absorption coefficient than the HDPE matrix and the best results take place using HDPE/GTR with two maximum values in 2000 and 5000 Hz. However, for EVA and PP composites the better noise reduction happens also for GTR reinforcement but at a frequency of 5500 Hz (Figure 12 and Figure 13).

To know why the composite reinforced with GTR has better acoustical properties than the composite reinforced with CFs, we can observe that the composite reinforced with GTR has more crazes, holes and is more porous than composites reinforced CFs. This is due mainly to the morphology of the reinforcement since GTR has an irregular polyhedron geometry with flat faces, straight edges and sharp corners, meanwhile CFs have a fibrous morphology with a smooth surface. This kind of morphology allowed the formation of holes, porous and crazes between them and between GTR and matrix. To explain this behavior, a model was used defined as “rigid-framed porous materials” [32]. This model considers that the cavity walls are non-deforming, and the increase in acoustic absorption takes place due to the viscous losses and thermo-elastic damping where the sound propagates through a large number of air cavities in the composite. The sound propagation is, therefore, governed by the effective density and effective bulk modulus of the air in the air spaces cavities. Very often it can be assumed that these composites are microporous materials, due mainly at the interphase area between thermoplastic matrix and GTR, where several microcavities appear around the surface of the GTR. When the volume of these cavities increases, the structure factor increases, resulting in a greater effective porosity, thereby increasing the maximum acoustic absorption.

## 4. Conclusions

Comparative analysis of different thermoplastic matrix composites reinforced with elastomeric and biogenic waste shows that: (i) the type of matrix is very important in determining the compatibility between the two components, seeing that EVA matrix defines better compatibility with both reinforced materials than polyolefin matrices (HDPE and PP); (ii) biogenic reinforcement (CFs) has better structural behavior than elastomeric reinforcement (GTR) due to higher compatibility of keratin (largely component of biogenic reinforced) with polyolephynic matrices, but mainly with EVA, as has been corroborated by FTIR and TGA; (iii) composites reinforced with GTR has better functional behavior (acoustic properties) than composites reinforced with CFs, due to the morphology of the reinforcing particles, where GTR defines an apparently irregular polyhedron geometry with a large number of microroughness in the surface that improves noise absorption when compared with the geometry of CFs particles (cylindrical, striated and very smooth).

The study of the behavior of two very different types of waste, with non-related chemical structures, biogenic and synthetic origins, dissimilar geometries and completely diverse properties shows that the incorporation of such residua in composites can be a successful approach to create materials suitable for specific applications, for construction, transport, entertainment and others. Nowadays, several proposals related to the use of fabricated composites, such as urban furniture, acoustic isolation panels, or non-structural panels for the automotive industry are under study. This path, leading to converting the subproducts of industry into useful products in other fields can be widened considering other matrices and the use of pretreatments, providing a way to fulfill the requirements of a circular economy concept.

## Data Availability

The data presented in this study are available on request from the corresponding author.
